# Article of Significant Interest in This Issue

**DOI:** 10.1128/iai.00409-25

**Published:** 2025-08-12

## BIOLUMINESCENT IMAGING TO INVESTIGATE *COXIELLA BURNETII* PATHOGENESIS IDENTIFIES ADIPOSE TISSUE AS A HOST NICHE FOR INFECTION

The human disease Q-fever is caused by the obligate intracellular bacterial pathogen *Coxiella burnetii*. Cell biological studies have defined the unique lysosome-derived vacuole in which *C. burnetii* replicates, but many questions remain as to how this pathogen spreads through the body to cause disease. Andrews and Roy (e00080-25) have used luminescent *C. burnetii* to investigate pathogenesis in a mouse model of infection. Bioluminescent imaging of whole animals revealed differences in the dissemination of *C. burnetii* and mutants defective in intracellular replication in this mouse model and identified host adipocytes as a preferred niche for bacterial replication. Understanding how *C. burnetii* targets adipocytes could provide important clues as to how this pathogen can persist and cause disease in mammalian hosts.

